# Supplementary material for: Monitoring benthic plumes, sediment redeposition and seafloor imprints caused by deep-sea polymetallic nodule mining
Source: Nat Commun. 2025 Jan 31;16:1229. doi: 10.1038/s41467-025-56311-0 (PMC11785793; doi:10.1038/s41467-025-56311-0)
Supplement: Supplementary file 1 — Supplementary Information [file 41467_2025_56311_MOESM1_ESM.pdf]

Supplementary Information

Monitoring benthic plumes, sediment redeposition and seafloor imprints caused by deep-sea polymetallic nodule mining

Iason-Zois Gazis[1*], Henko de Stigter[2], Jochen Mohrmann[1], Karl Heger[1], Melanie Diaz[2], Benjamin Gillard[3], Matthias Baeye[4], Mario E. Veloso-Alarcón[1], Kaveh Purkiani[1,5,6], Matthias Haeckel[1], Annemiek Vink[7], Laurenz Thomsen[3,8], Jens Greinert[1,9]

[1] GEOMAR Helmholtz Centre for Ocean Research Kiel, Germany

[2] NIOZ Royal Netherlands Institute for Sea Research, Texel, The Netherlands

[3] Constructor University, Bremen, Germany

[4] Royal Belgian Institute of Natural Sciences, Brussels, Belgium

[5] MARUM Center for Marine Environmental Sciences and Faculty of Geosciences, University of Bremen, Bremen, Germany

[6] Now at Federal Maritime and Hydrographic Agency, Hamburg, Germany

[7] Federal Institute for Geosciences and Natural Resources, Hannover, Germany

[8] Now at the University of Gothenburg, Sweden

[9] Institute of Geosciences, Christian-Albrecht University of Kiel, Germany

[*] *Corresponding author: igazis@geomar.de*

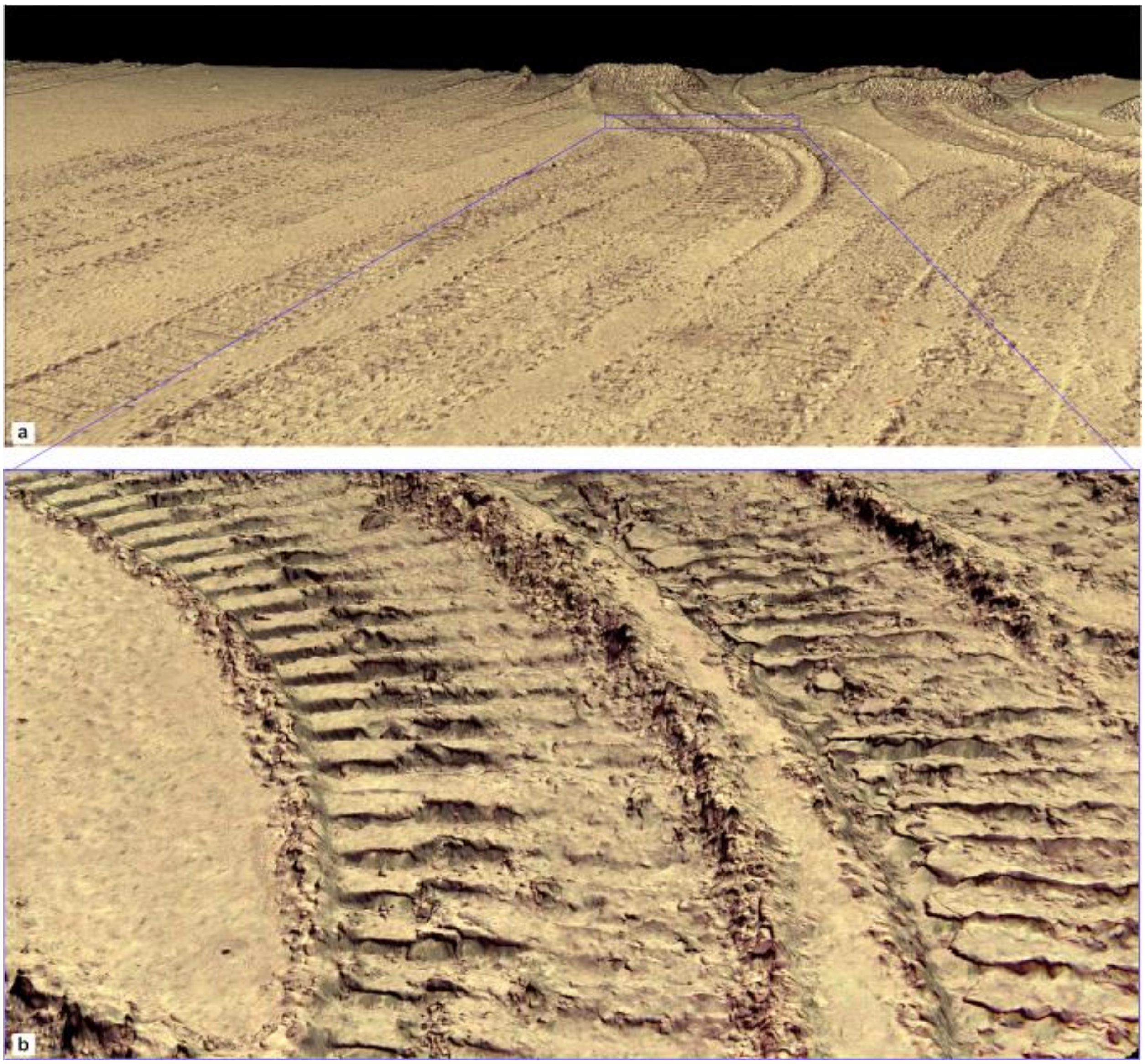

Fig: S1 | 3D orthophoto-mosaic of the nodule collector trial site.

a) 3D perspective view from an oblique angle of the caterpillar track imprints along the mining lanes (part of the second strip, as presented in Fig. 2). The redeposited sediment covers the unmined seafloor between the mining lanes and only the humps of the unmined nodules are partly visible. **b)** Close-up of the small-scale seafloor morphology (cm scale) created by the nodule collector caterpillar tracks along the "light bulb" turning maneuver shortly before the deposition of the nodule pile at the line end. These imprints are locally deeper (up to 8 cm) than those made along the mining lanes due to the additional nodule payload (~ 3 t). The two caterpillar imprints have a total length of 4 m and average depth of 5 cm.

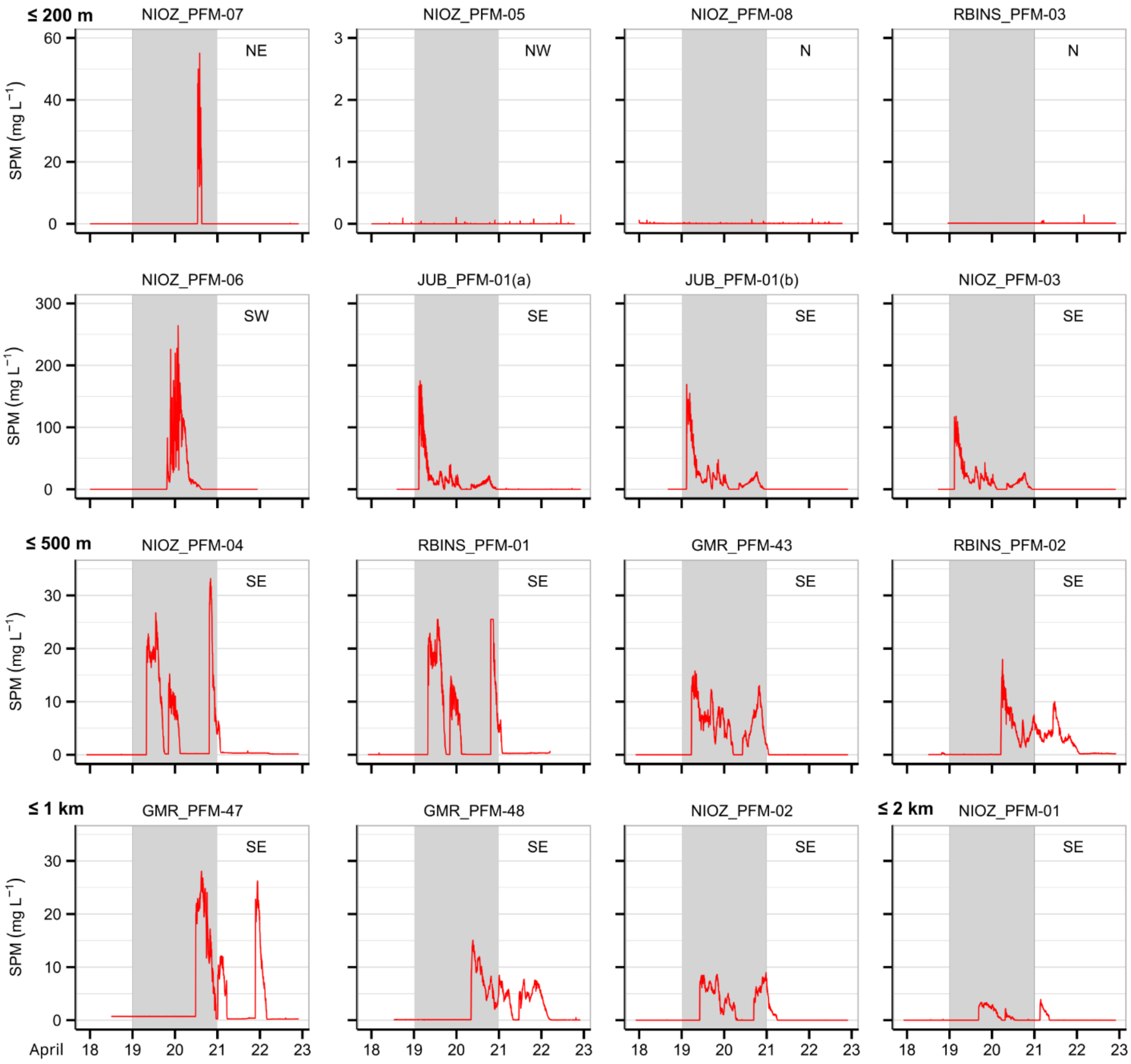

Fig. S2 | Time-series of suspended particulate matter concentration

The optical backscatter sensors (OBS) recorded the suspended particulate matter (SPM) concentrations 1 m above the seafloor. The seafloor platforms have been grouped by distance and orientation to the impact site. The grey sections indicate the nodule collector operation time (from 19 April 2021 00:52 UTC to 20 April 2021 17:25 UTC).

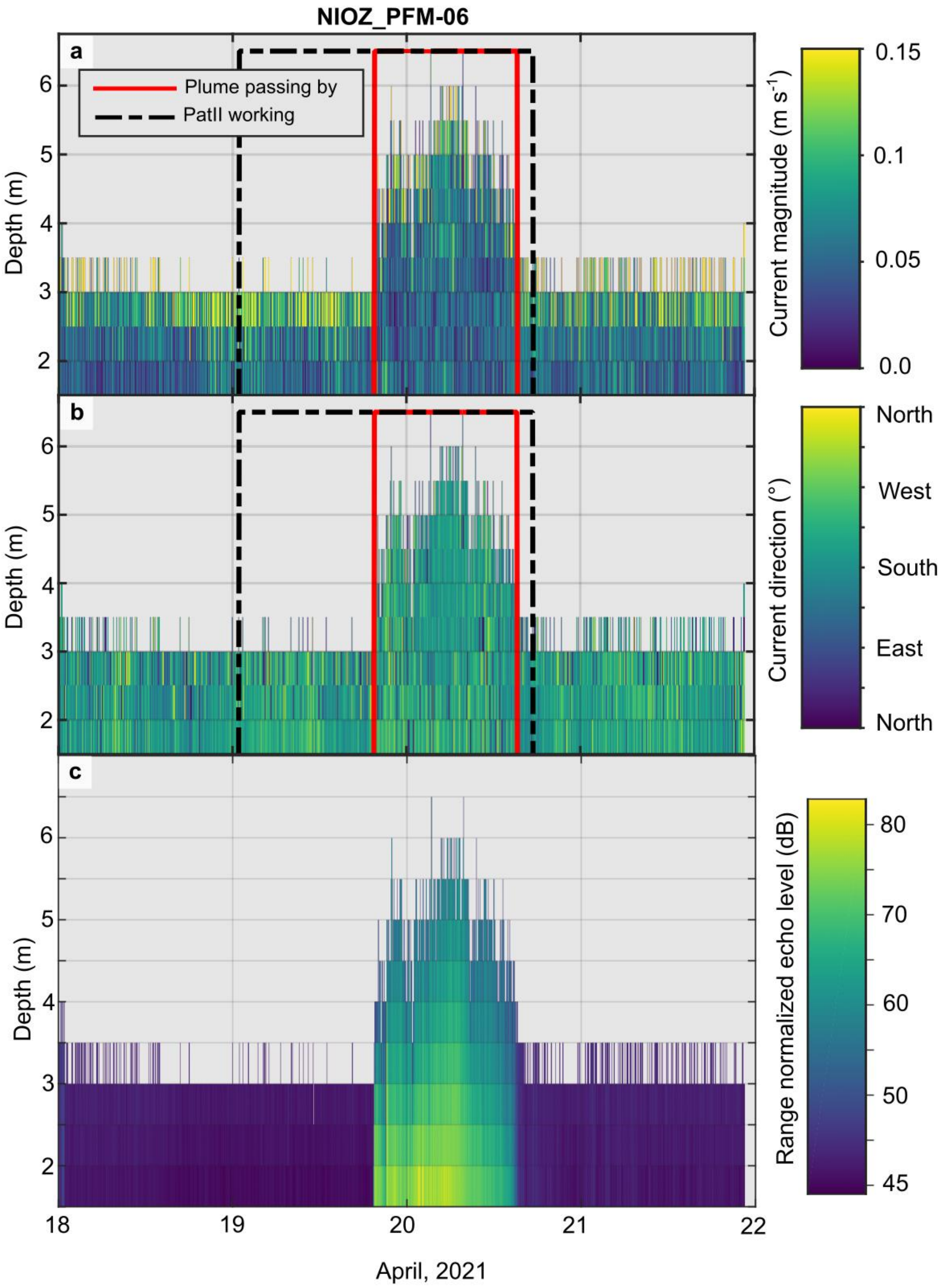

Fig. S3 | Acoustic doppler current profiler (ADCP) data at the NIOZ_PFM-06 platform.

a) Current magnitude, **b)** current direction and **c)** range normalized backscatter intensity at the NIOZ_PFM-06 platform between 18 and 22 April 2021. The nodule collector (PatII) operation time and the passage of the benthic sediment plume are indicated in Figures a and b.

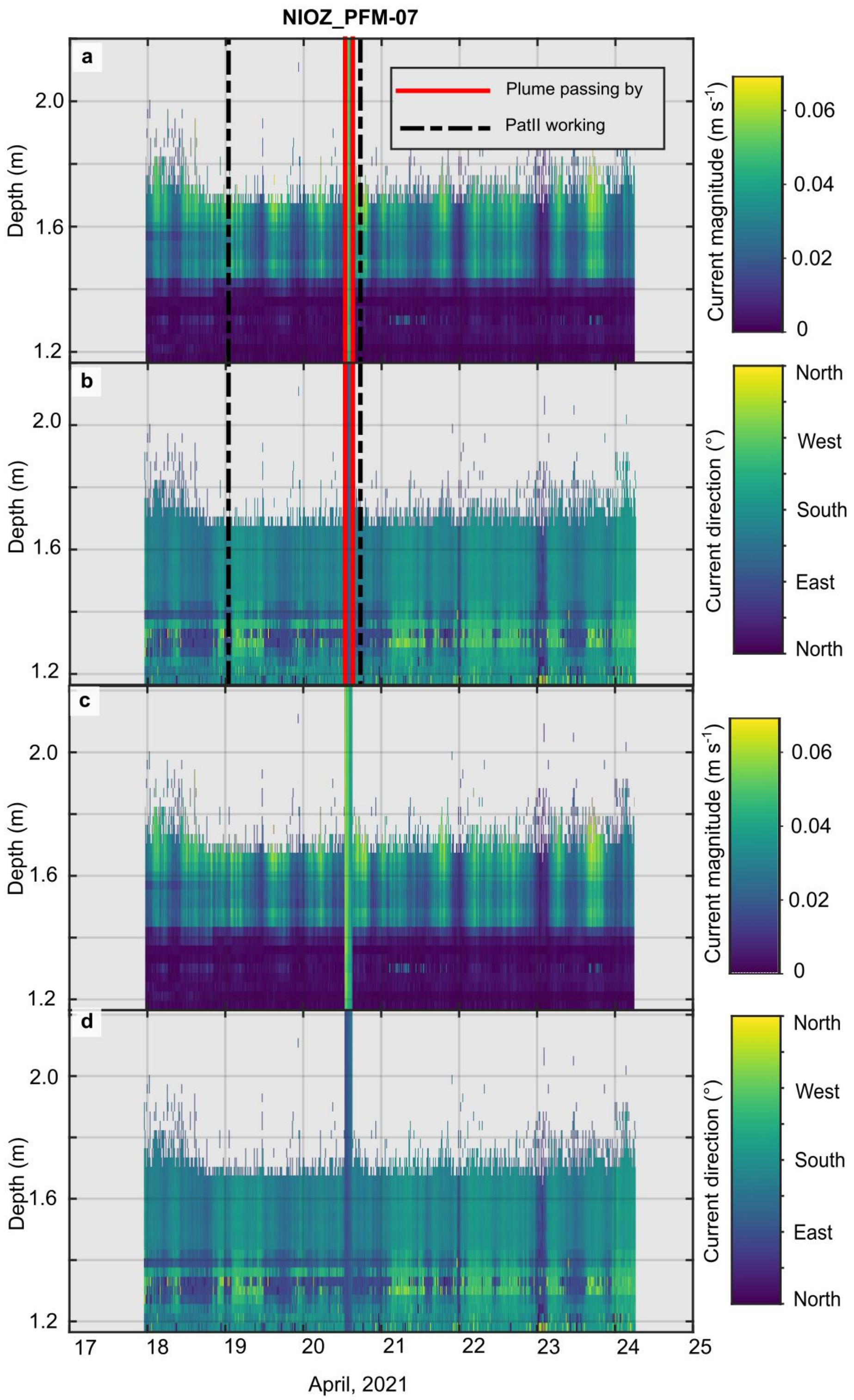

Fig. S4 | Acoustic doppler current profiler (ADCP) data at the NIOZ_PFM-07 platform. Current magnitude, **b)** current direction and **c)** range normalized backscatter intensity at the NIOZ_PFM-07 platform between 18 and 25 April 2021, with (a and b) and without (c and d) the nodule collector (PatII) activity and benthic sediment plume passage time. The gravity current created a distinct change in current speed and direction.

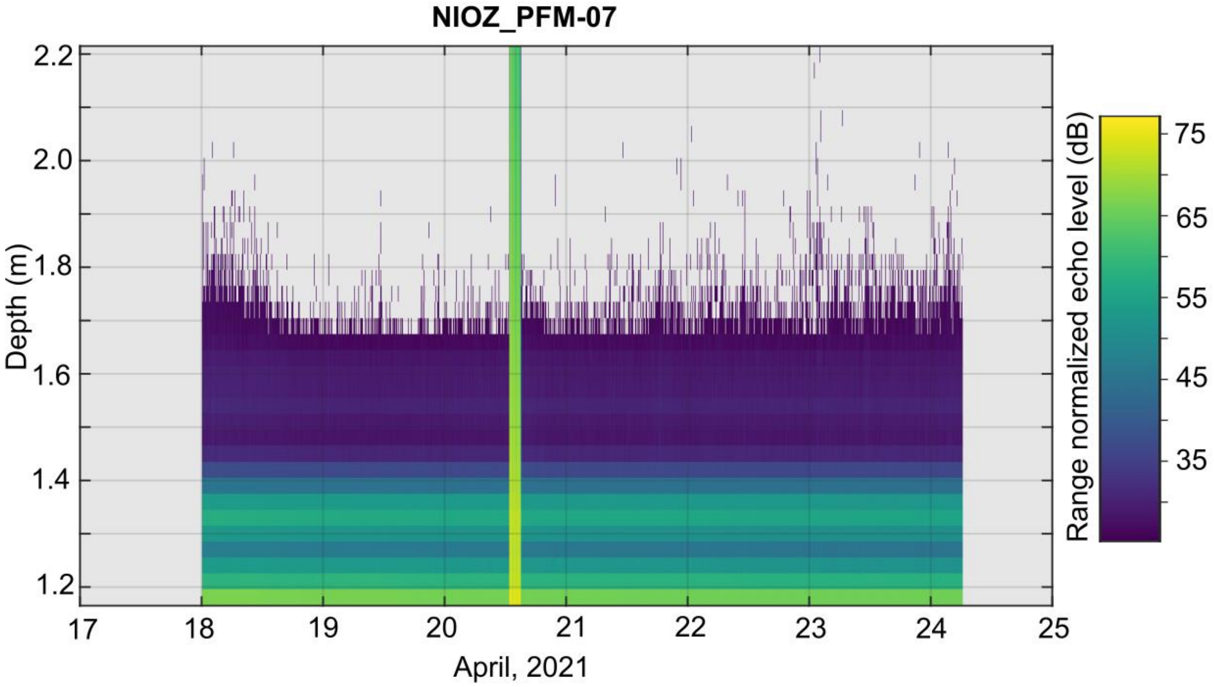

Fig. S5 | Range normalized water-column backscatter at the NIOZ_PFM-07 platform.

Range normalized water-column backscatter intensity from the upward-looking ADV (2MHz) at the NIOZ_PFM-07 platform between 18 and 25 April 2021, showing the gravity current's passage.

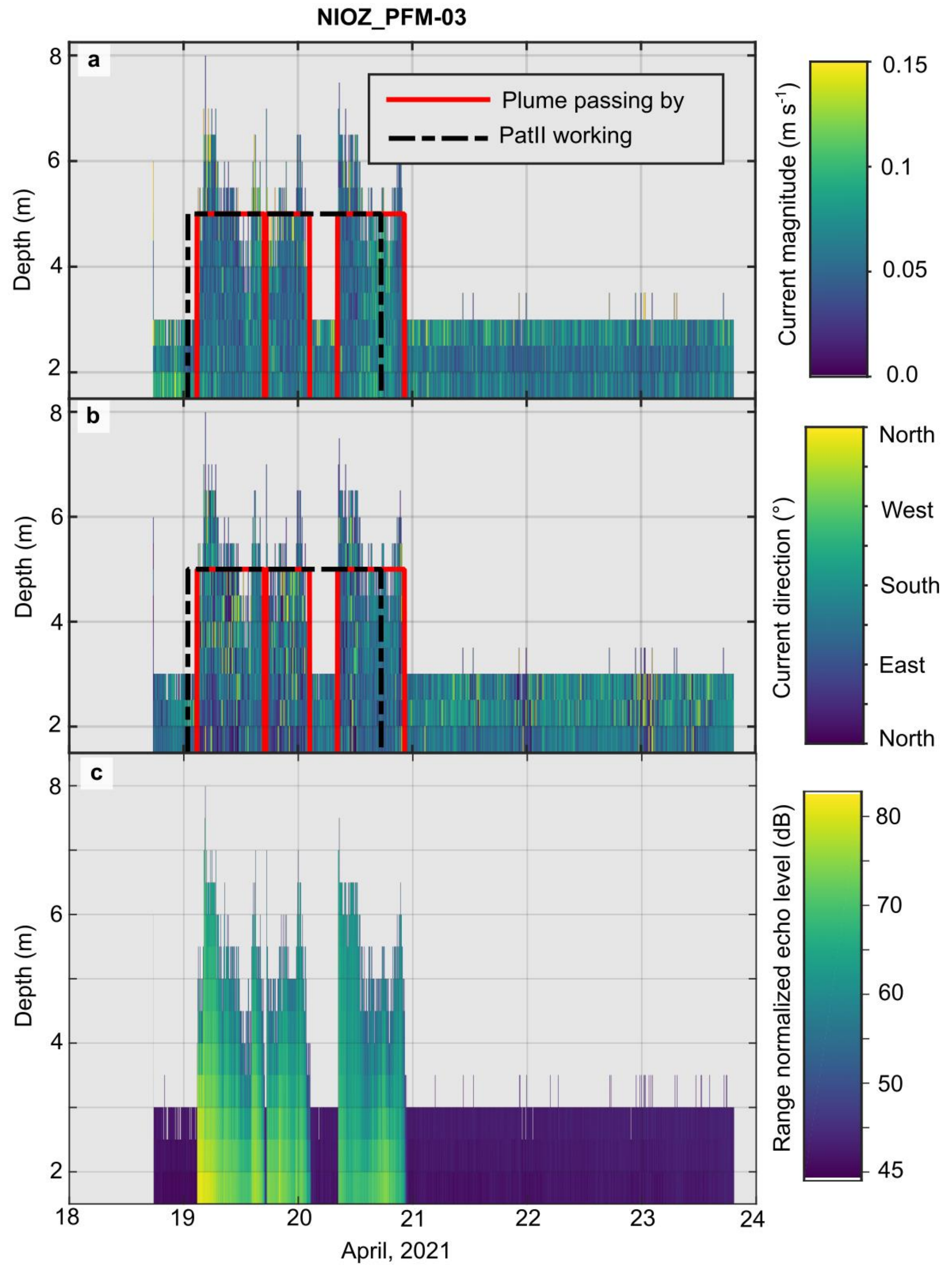

Fig. S6 | Acoustic doppler current profiler (ADCP) data at the NIOZ_PFM-03 platform.

a) Current magnitude, **b)** current direction, and **c)** range normalized backscatter intensity from the ADCP (2 MHz) at the NIOZ_PFM-03 platform between 18 April and 24 April 2021. The nodule collector (PatII) operation time and the passage of the benthic sediment plume are indicated in Figures a and b.

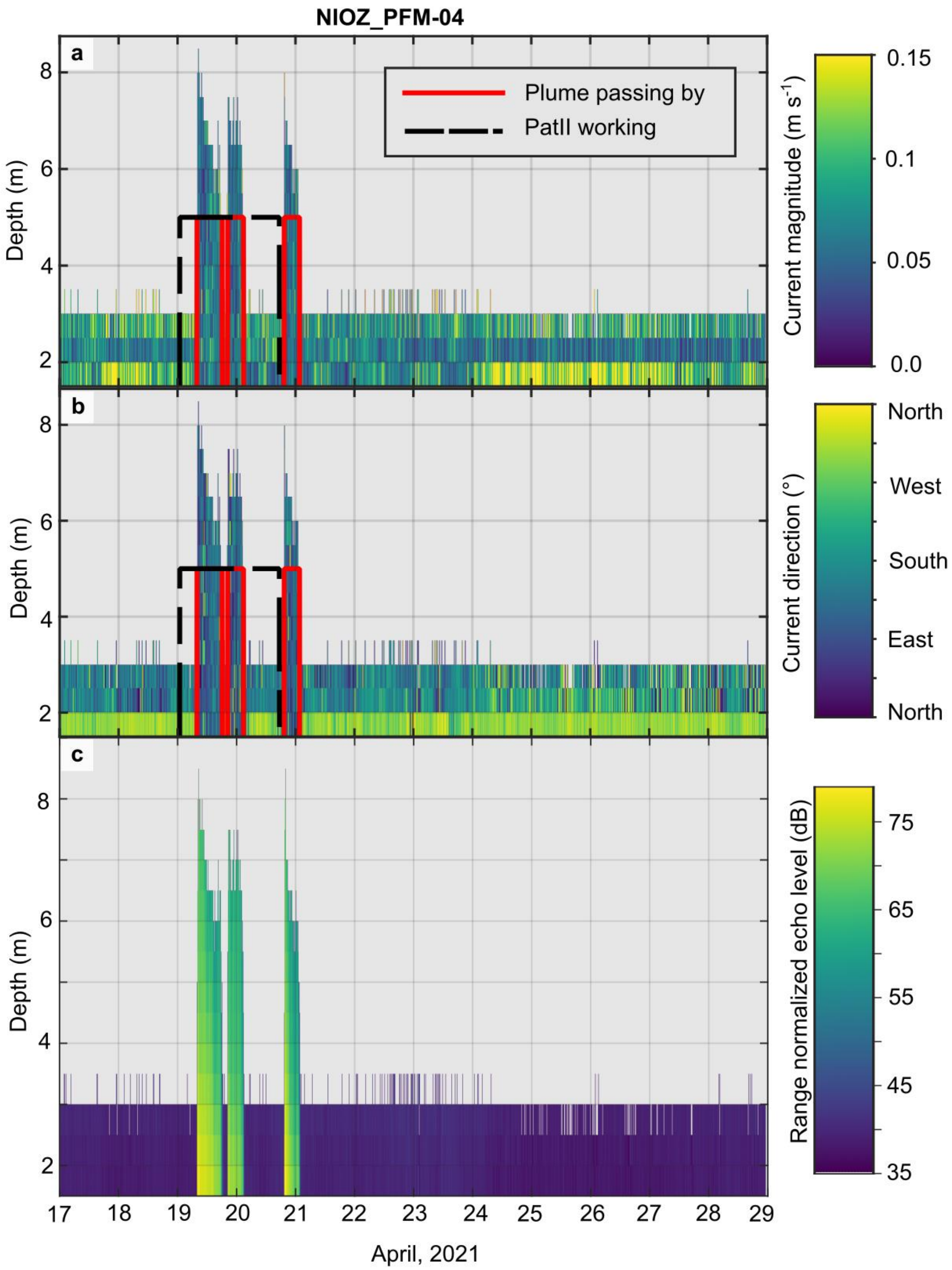

Fig. S7 | Acoustic doppler current profiler (ADCP) data at the NIOZ_PFM-04 platform.

a) Current magnitude, **b)** current direction and **c)** range normalized backscatter intensity from the upward-looking ADCP (2 MHz) at the NIOZ_PFM-04 platform between 17 and 29 April 2021. The ADCP at platform NIOZ_PFM-04 recorded the most distant change in the current direction when the benthic sediment plume passed. The nodule collector (PatII) operation time and the passage of the benthic sediment plume are indicated in Figures a and b.

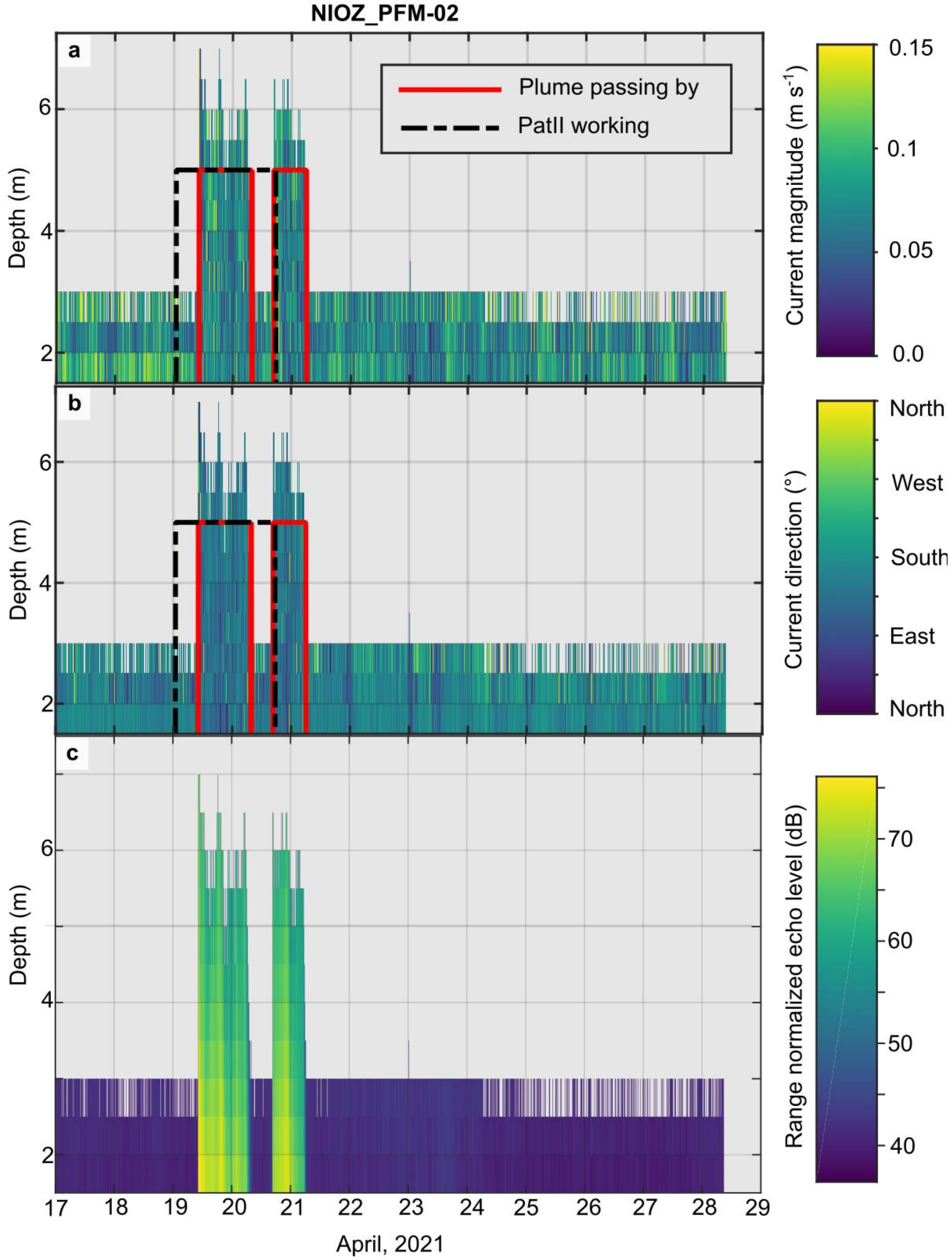

Fig. S8 | Acoustic doppler current profiler (ADCP) data at the NIOZ_PFM-02 platform.

a) Current magnitude, **b)** current direction and **c)** range normalized backscatter intensity from the upward-looking ADCP (2 MHz) at the NIOZ_PFM-02 platform between 17 and 29 April 2021. At 1 km SE from the impact site, the benthic sediment plume has the same direction as the ambient bottom currents. The nodule collector (PatII) operation time and the passage of the benthic sediment plume are indicated in Figures a and b.

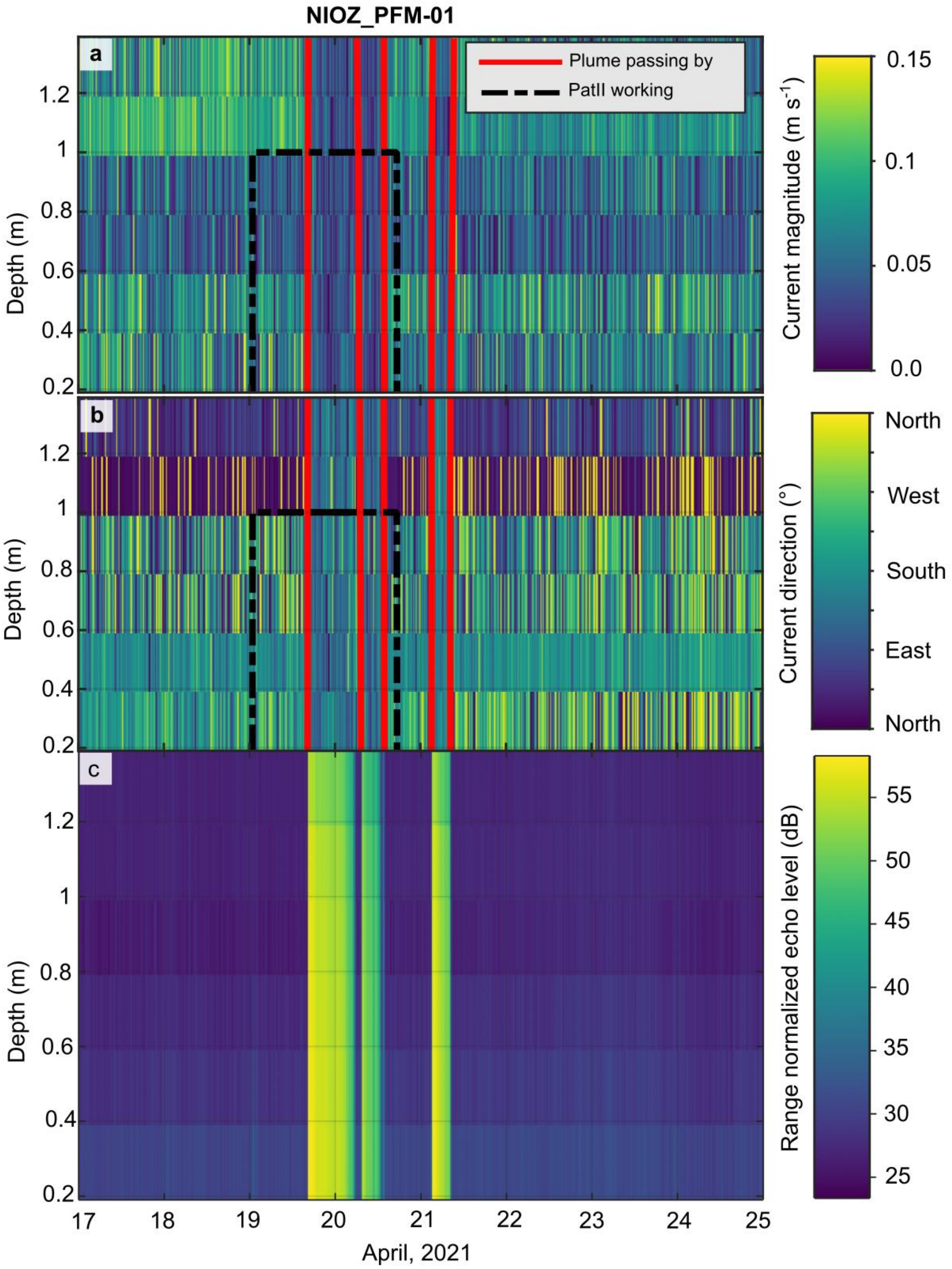

Fig. S9 | Acoustic doppler current profiler (ADCP) downward-looking data at the NIOZ_PFM-01 platform. **a)** Current magnitude, **b)** current direction and **c)** range normalized backscatter intensity from the downward-looking ADCP (1.2 MHz) at the NIOZ_PFM-01 platform between 17 April and 25 April 2021. The most remote downward-looking ADCP showed that the benthic sediment plume moves in the SE direction. The nodule collector (PatII) operation time and the passage of the benthic sediment plume are indicated in Figures a and b.

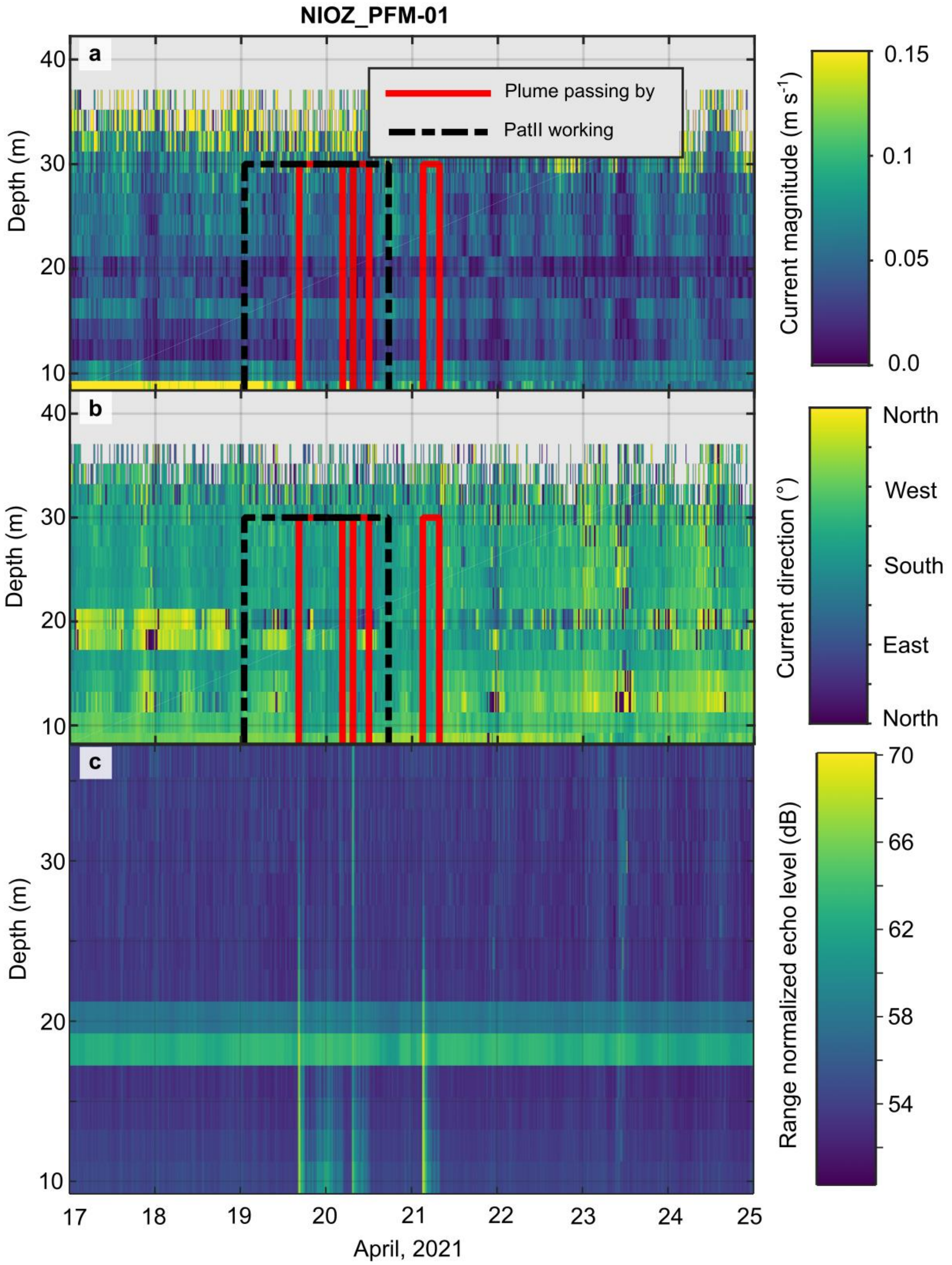

Fig. S10 | Acoustic doppler current profiler (ADCP) upward-looking data at the NIOZ_PFM-01 platform. a) Current magnitude, b) current direction and c) range normalized backscatter intensity from the upward-looking ADCP (300 kHz) at the NIOZ_PFM-01 platform between 17 April and 25th April 2021. The most remote upward-looking ADCP at 1800m SE from the impact site recorded the suspended particles at 30+ m altitude. The nodule collector (PatII) operation time and the passage of the benthic sediment plume are indicated in Figures a and b.

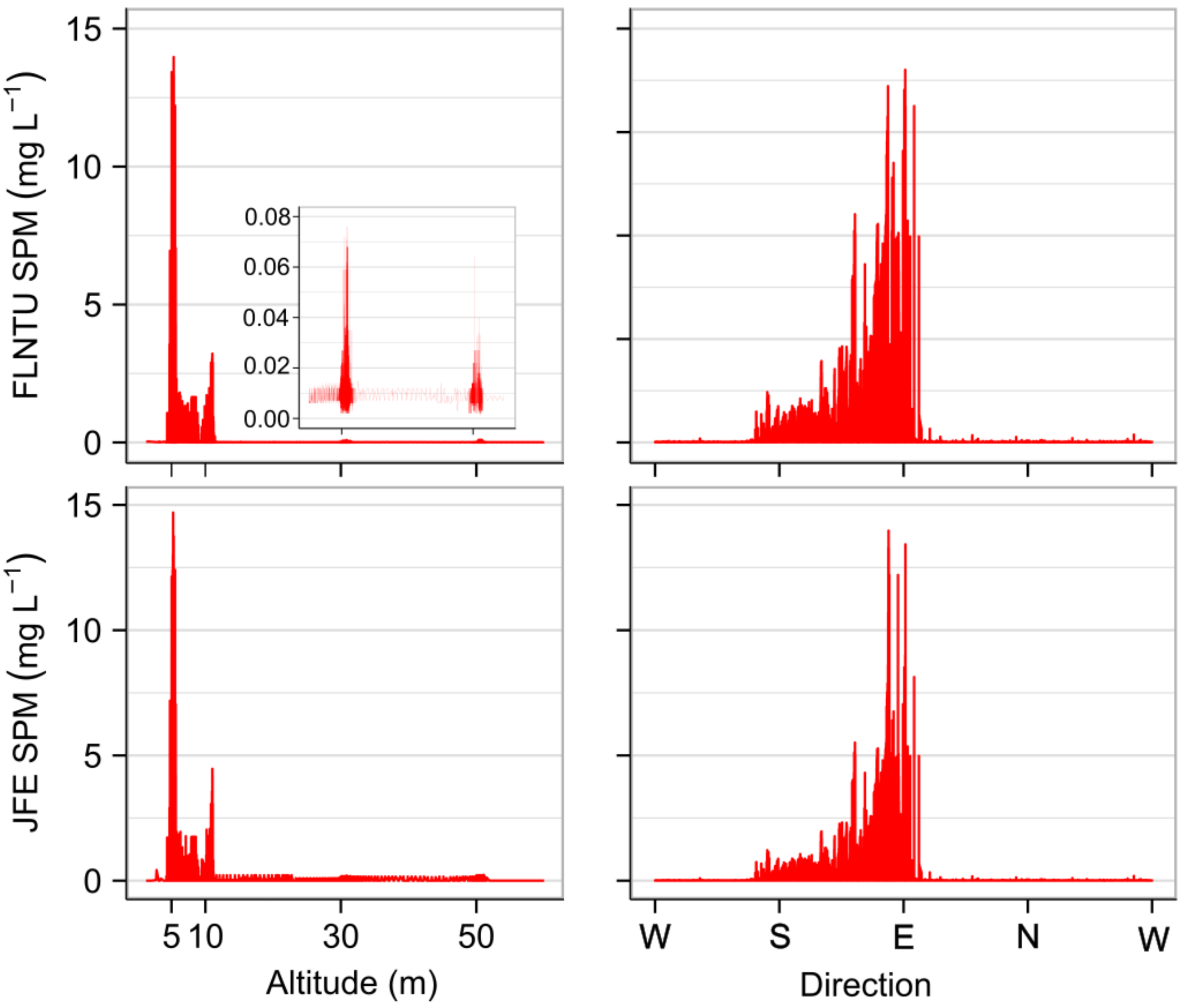

Fig. S11 | Suspended particulate matter (SPM) concentration recorded by the autonomous underwater vehicle (AUV)

The AUV conducted a ca. 54 h survey parallel to the mining trial and immediately after recording the SPM concentration with altitude and direction with two different optical backscatter sensors (OBS), namely FLNTU and JFE (see Methods). **a-b)** FLNTU SPM concentration for different monitoring altitudes and directions, **c-d)** JFE SPM concentration for different monitoring altitudes and directions. The data clearly show that the benthic sediment plume spread in the S to E direction and that the highest concentrations were registered at 5 m, which was the lowest monitoring altitude by the AUV. Even the highest peak in SPM concentration is less than half of the SPM concentration recorded at 1 m altitude at the same distance and direction from the impact site (Fig. S29).

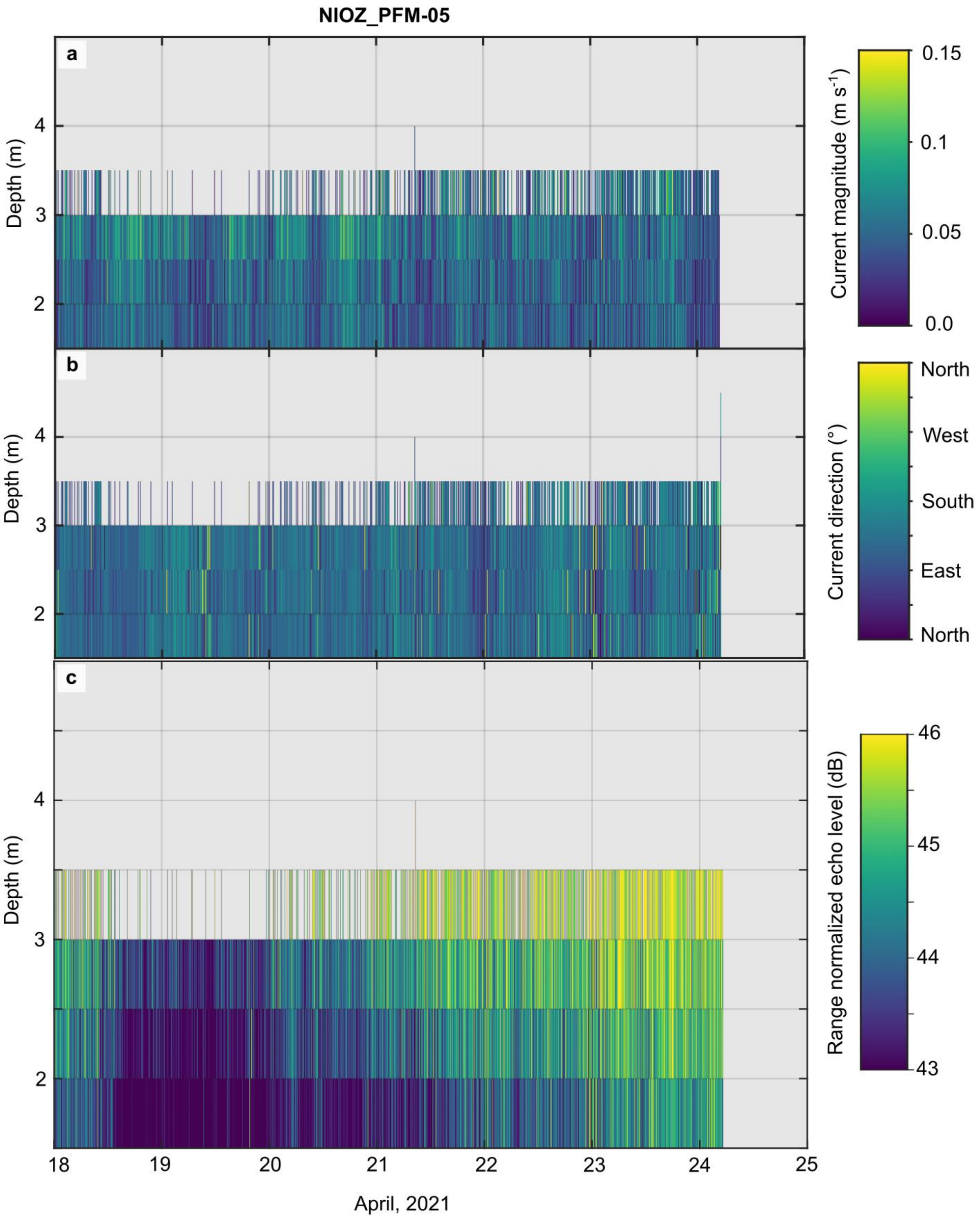

Fig. S12 | Acoustic doppler current profiler (ADCP) data at the NIOZ_PFM-05 platform.

a) Current magnitude, **b)** current direction, and **c)** range normalized backscatter intensity from the upward-looking ADV (2 MHz) at the NIOZ_PFM-05 platform between 18 April and 25April 2021. The benthic sediment plume was not detected NW of the impact site.

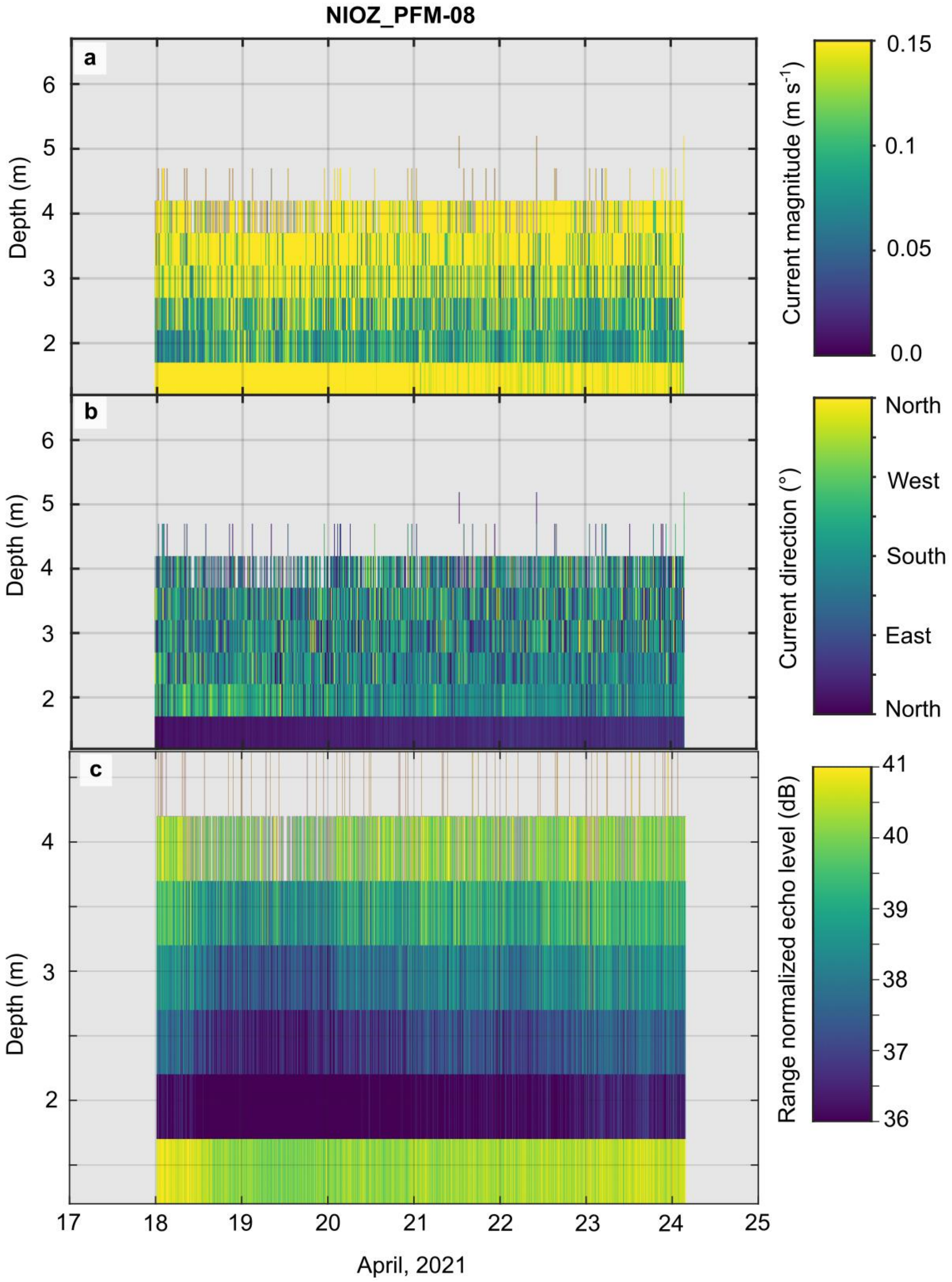

Fig. S13 | Acoustic doppler current profiler (ADCP) data at the NIOZ_PFM-08 platform.

a) Current magnitude, **b)** current direction, and **c)** range normalized backscatter intensity from the upward-looking ADV (2 MHz) at the NIOZ_PFM-08 platform between 18 April and 24 April 2021. The benthic sediment plume was not detected at the N of the impact site.

Table S1 | Summary statistics of the particle size (D_{25}, D_{50}, D_{75}) for each benthic sediment plume episode (plume).

1st Plume Episode	Min.	1st Qu.	Median	Mean	3rd Qu.	Max.
D_{25} (raw)	59.0	69.7	73.0	74.7	77.0	101.0
D_{25} (after roll median)	61.0	70.0	73.0	74.2	77.0	97.0
D_{50} (raw)	86.0	99.7	107.0	110.5	118.0	150.00
D_{50} (after roll median)	86.0	102.0	108.0	109.3	117.0	145.00
D_{75} (raw)	115.0	139.0	148.5	164.9	175.0	473.0
D_{75} (after roll median)	124.00	143.2	149.5	155.7	170.0	202.0
2nd Plume Episode	Min.	1st Qu.	Median	Mean	3rd Qu.	Max.
D_{25} (raw)	62.0	90.5	104.0	100.6	114.0	129.0
D_{25} (after roll median)	68.0	92.5	106.0	101.0	114.0	122.0
D_{50} (raw)	78.0	129.2	147.5	142.9	159.8	187.0
D_{50} (after roll median)	97.0	130.5	148.5	143.5	159.0	169.0
D_{75} (raw)	93.0	172.5	195.0	190.6	209.8	267.0
D_{75} (after roll median)	130.0	174.0	195.0	189.8	209.0	246.0
3rd Plume Episode	Min.	1st Qu.	Median	Mean	3rd Qu.	Max.
D_{25} (raw)	66.0	82.0	97.0	98.6	116.0	152.0
D_{25} (after roll median)	68.0	82.0	101.0	98.5	116.0	140.0
D_{50} (raw)	95.0	123.0	146.5	144.6	165.5	218.0
D_{50} (after roll median)	100.0	123.0	147.0	142.0	164.0	200.0
D_{75} (raw)	130	178.5	200.0	210.0	229.0	487.0
D_{75} (after roll median)	140	177.0	200.0	199.6	218.0	313.0

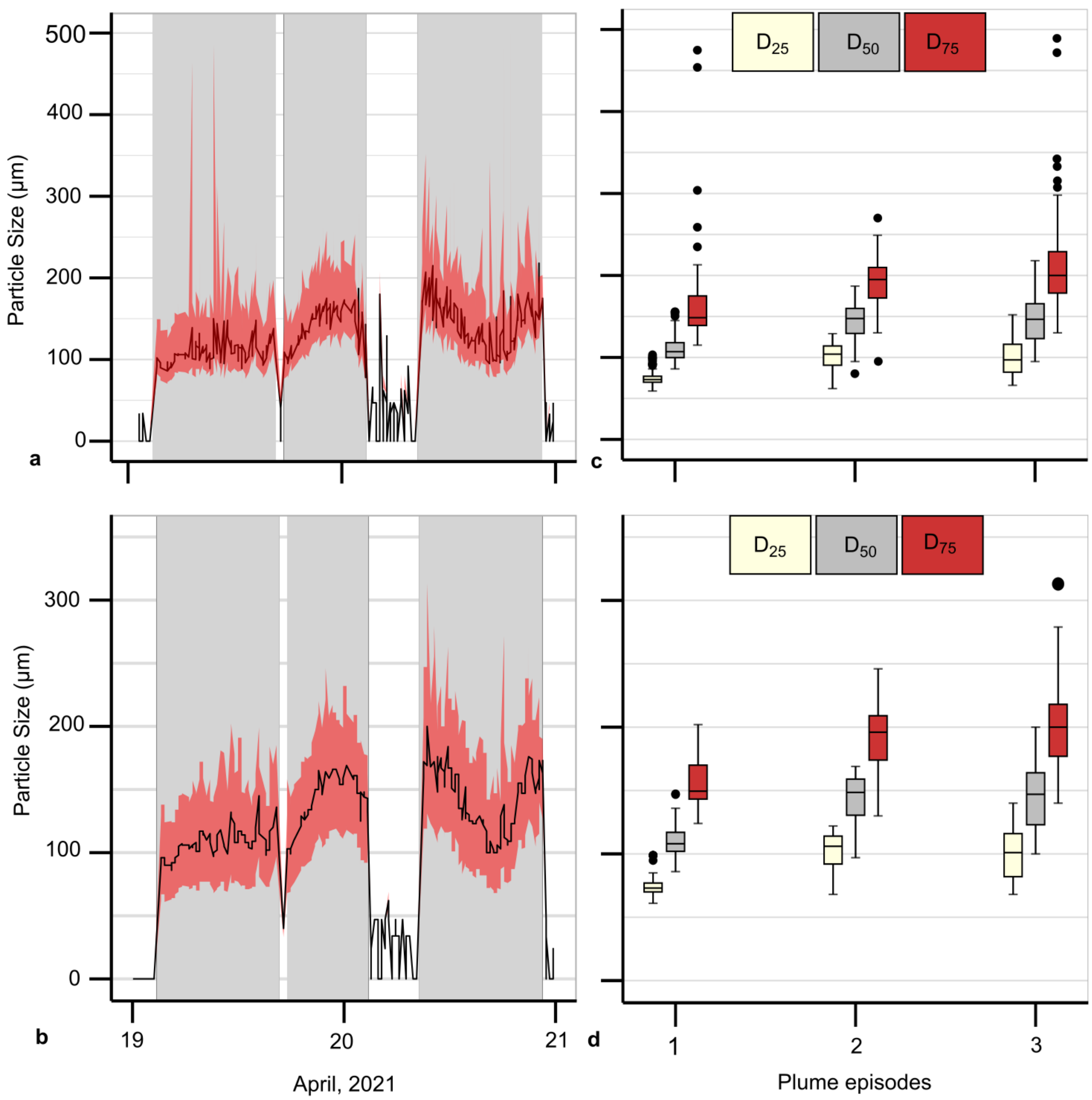

Fig. S14 | Particle number and size before and after applying a rolling median

a) Time series of particle size (raw data) during the three benthic sediment plume episodes, which are shown in grey. The black line is the D_{50}, and the red area shows the upper D_{75} and lower D_{25} of the particle size distribution. Few isolated outliers are observed in raw data. **b)** Same as a, after applying a rolling median (k=3, with k being the integer width of the rolling window). **c)** Boxplots of the particle sizes D_{25}, D_{50} and D_{75} for the fisrt (1), second (2) and third (3) plume episodes using the raw data (n=258 observations). **d)** Same as c, after applying a rolling median (k=3). Both in c and d the black centre line is the median value (50th percentile), and the boxes contain each dataset's 25th to 75th percentiles. The black whiskers mark the 5th and 95th percentiles, and values beyond these bounds are outliers (black dots).

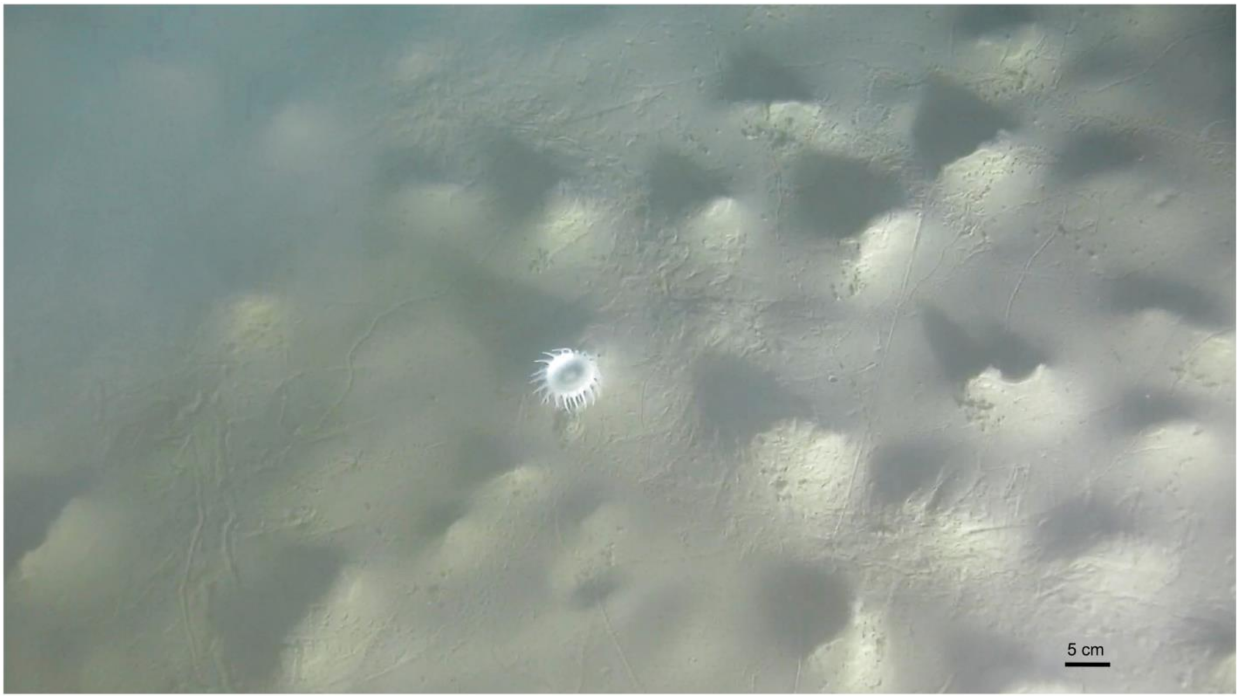

Fig. S15 | Seafloor image inside the mining site

A seafloor image obtained by the remotely operated vehicle 5 m from a mining lane shows that redeposited sediment completely covers nodules (sediment blanketing). A sea anemone (*Actiniaria*) was recorded on the redeposited sediment. The image location is shown in Fig. 10.

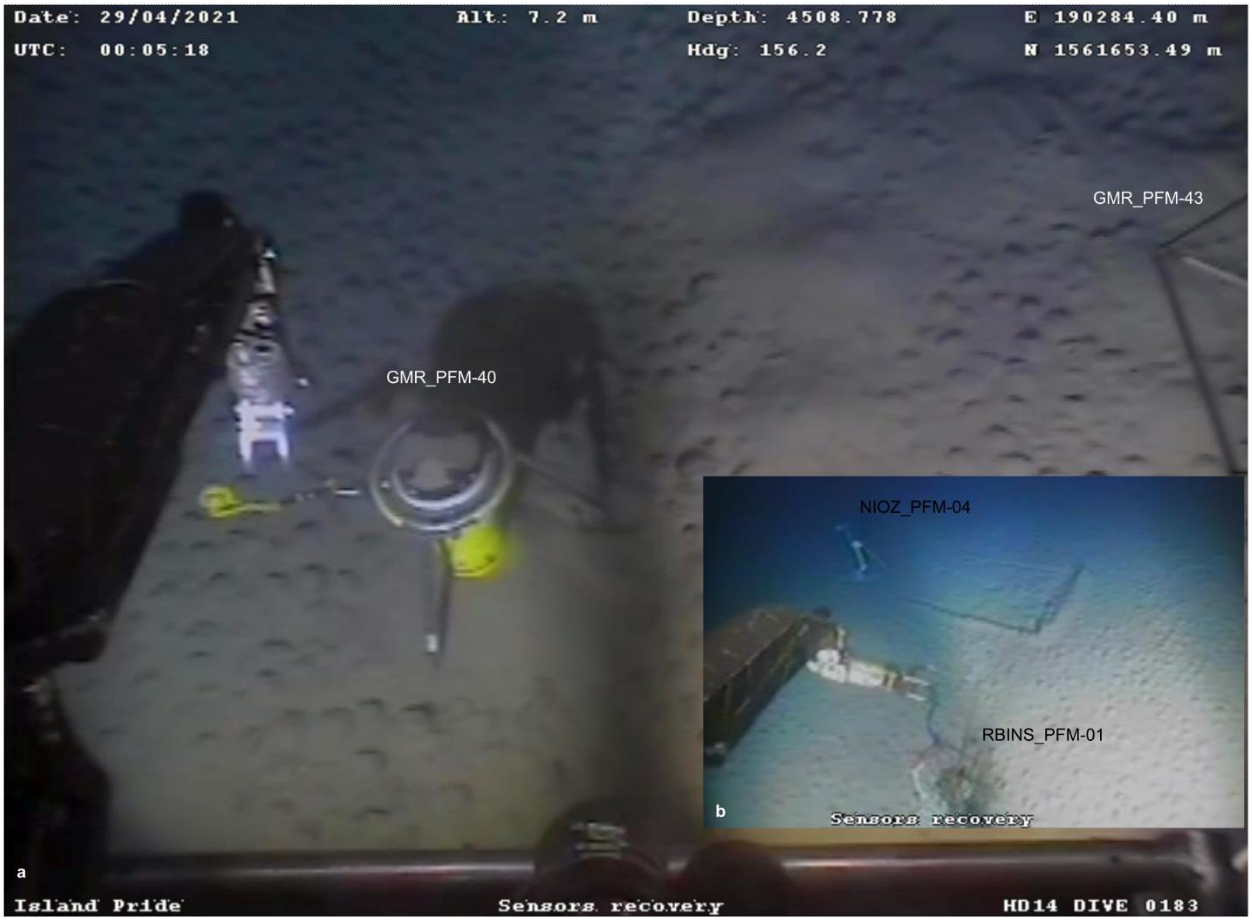

Fig. S16 | Sediment redeposition 500 m E-SE from the impact site.

Video frame grab shows the seafloor, GMR_PFM-40, and GMR_PFM-43 platforms at 500 SE from the impact site. The distance between A and B is 0.75 m. **b)** Video frame grab that shows the seafloor and NIOZ_PFM-04 and RBINS_PFM-01 platforms at 500 m E from the impact site. In both images, the redeposited sediment has covered the nodules. Please see Fig. 3 for the location of the platforms.

a		Pre-impact	Post-impact
Distance from mining lanes (m)	50	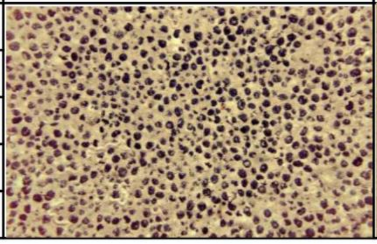	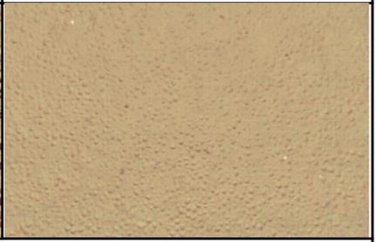
Distance from nearest OBS (m)	0.15		
Maximum SPM (mg L^{-1})	264		
Pre-detectable coverage (%)	44		
Post-detectable coverage (%)	1.7		
b			
Distance from mining lanes (m)	50	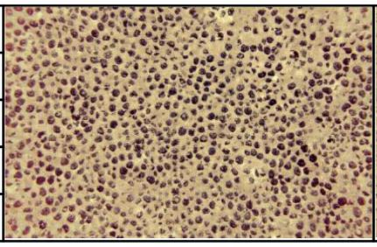	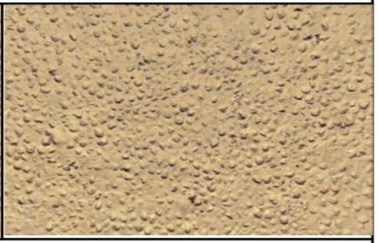
Distance from nearest OBS (m)	200		
Maximum SPM (mg L^{-1})	35		
Pre-detectable coverage (%)	27.5		
Post-detectable coverage (%)	10.5		
c			
Distance from mining lanes (m)	1000	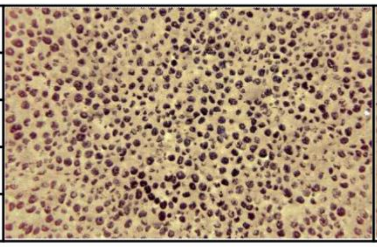	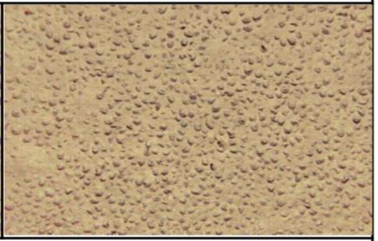
Distance from nearest OBS (m)	160		
Maximum SPM (mg L^{-1})	28		
Pre-detectable coverage (%)	27.1		
Post-detectable coverage (%)	9.5		
d			
Distance from mining lanes (m)	1800	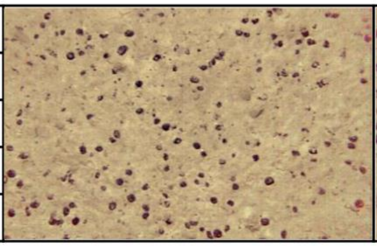	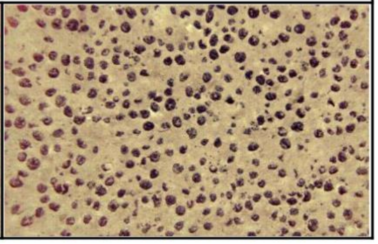
Distance from nearest OBS (m)	300		
Maximum SPM (mg L^{-1})	3.9		
Pre-detectable coverage (%)	6.5		
Post-detectable coverage (%)	20.1		

Fig. S17 | Seafloor images before and after the sediment redeposition

a) 50 m W from the impact site (next to the NIOZ_PFM-06 platform). **b-d)** 500–1800 m SE from the impact site along the AUV survey lines shown in Figure 10. **d)** The images are at the end of the survey lines outside the zone of sediment redeposition (see Fig.10). The lower nodule coverage in the pre-impact image is due to the seafloor spatial variability in nodule coverage; the pre-impact image was taken within a depression that has lower nodule coverage[1]. The lateral distance from the nearest optical backscatter sensor (OBS), the maximum suspended particulate matter (SPM) concentration and the % detectable seafloor nodule coverage per square meter (see Methods) are provided.

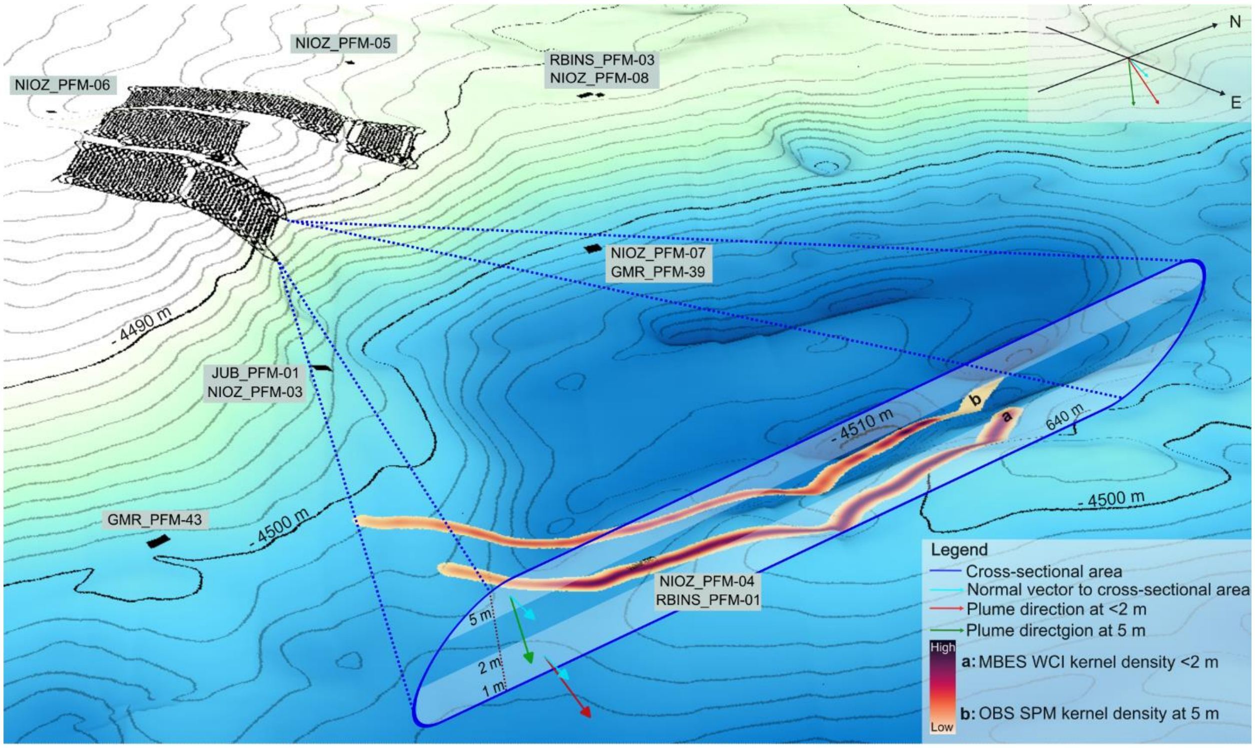

Fig. S18 | Cross-sectional area of the benthic sediment plume for the first 5 m altitude

Schematic representation of the cross-sectional area of the benthic sediment plume, generated during the first mining strip. The multibeam echosounder water-column imaging (WCI) and the suspended particulate matter (SPM) concentrations recorded by the optical backscatter sensors (OBS) mounted on the autonomous underwater vehicle have the same lateral extent. The cross-section is located 500 m E-SE from the impact site, it has a height of 5 m and a length of 640 m. The seafloor vertical exaggeration is × 8. The location of seafloor platforms is also given.

References

[1] Gazis, I.-Z., Charlet, F., & Greinert, J. An interpretable multi-model machine learning approach for spatial mapping of deep-sea polymetallic nodule occurrences. *Natural Resources Research*. (2024).
