# Supplementary material for: Monitoring benthic plumes, sediment redeposition and seafloor imprints caused by deep-sea polymetallic nodule mining
Source: Nat Commun. 2025 Jan 31;16:1229. doi: 10.1038/s41467-025-56311-0 (PMC11785793; doi:10.1038/s41467-025-56311-0)
Supplement: Supplementary file 3 — Reporting Summary [file 41467_2025_56311_MOESM3_ESM.pdf]

nature portfolio

Corresponding author(s): Iason-Zois Gazis

Last updated by author(s): Dec 10, 2024

Reporting Summary

Nature Portfolio wishes to improve the reproducibility of the work that we publish. This form provides structure for consistency and transparency in reporting. For further information on Nature Portfolio policies, see our Editorial Policies and the Editorial Policy Checklist.

Statistics

For all statistical analyses, confirm that the following items are present in the figure legend, table legend, main text, or Methods section.

n/a	Confirmed	
☒	☐	The exact sample size (*n*) for each experimental group/condition, given as a discrete number and unit of measurement
☒	☐	A statement on whether measurements were taken from distinct samples or whether the same sample was measured repeatedly
☒	☐	The statistical test(s) used AND whether they are one- or two-sided<br>*Only common tests should be described solely by name; describe more complex techniques in the Methods section.*
☒	☐	A description of all covariates tested
☒	☐	A description of any assumptions or corrections, such as tests of normality and adjustment for multiple comparisons
☒	☐	A full description of the statistical parameters including central tendency (e.g. means) or other basic estimates (e.g. regression coefficient) AND variation (e.g. standard deviation) or associated estimates of uncertainty (e.g. confidence intervals)
☒	☐	For null hypothesis testing, the test statistic (e.g. *F*, *t*, *r*) with confidence intervals, effect sizes, degrees of freedom and *P* value noted<br>*Give P values as exact values whenever suitable.*
☒	☐	For Bayesian analysis, information on the choice of priors and Markov chain Monte Carlo settings
☒	☐	For hierarchical and complex designs, identification of the appropriate level for tests and full reporting of outcomes
☒	☐	Estimates of effect sizes (e.g. Cohen's *d*, Pearson's *r*), indicating how they were calculated

Our web collection on statistics for biologists contains articles on many of the points above.

Software and code

Policy information about availability of computer code

Data collection	Field work exclusively: Research cruise onboard MV Island Pride.
Data analysis	No new software or custom algorithm was developed. All software and algorithms used, are cited in the manuscript.

For manuscripts utilizing custom algorithms or software that are central to the research but not yet described in published literature, software must be made available to editors and reviewers. We strongly encourage code deposition in a community repository (e.g. GitHub). See the Nature Portfolio guidelines for submitting code & software for further information.

Data

Policy information about availability of data

All manuscripts must include a data availability statement. This statement should provide the following information, where applicable:

- Accession codes, unique identifiers, or web links for publicly available datasets
- A description of any restrictions on data availability
- For clinical datasets or third party data, please ensure that the statement adheres to our policy

All data associated with the results of this study are presented in the paper, supplementary material and online video content. The data will be published on PANGEA Data Publisher for Earth & Environmental Science and can be obtained from the corresponding author upon reasonable personal request.

Research involving human participants, their data, or biological material

Policy information about studies with human participants or human data. See also policy information about sex, gender (identity/presentation), and sexual orientation and race, ethnicity and racism.

Reporting on sex and gender	n/a
Reporting on race, ethnicity, or other socially relevant groupings	n/a
Population characteristics	n/a
Recruitment	n/a
Ethics oversight	n/a

Note that full information on the approval of the study protocol must also be provided in the manuscript.

Field-specific reporting

Please select the one below that is the best fit for your research. If you are not sure, read the appropriate sections before making your selection.

☐ Life sciences ☐ Behavioural & social sciences ☒ Ecological, evolutionary & environmental sciences

For a reference copy of the document with all sections, see nature.com/documents/nr-reporting-summary-flat.pdf

Ecological, evolutionary & environmental sciences study design

All studies must disclose on these points even when the disclosure is negative.

Study description	This study describes the scientific monitoring of a deep-sea trial of a pre-prototype polymetallic nodule collector.
Research sample	Hydroacoustic and optic backscatter data.
Sampling strategy	Field work exclusively: Research cruise onboard MV Island Pride.
Data collection	Deployment of oceanographic sensors, remotely operated vehicles (ROVs) and autonomous underwater vehicle (AUV).
Timing and spatial scale	The data collection took place from 18.04.2021 to 25.04.2021. Data was collected from 50 m to 5 km from the source.
Data exclusions	No data excluded.
Reproducibility	Multiple similar sensors were mounted on each stationary seafloor platform and moving platforms (ROVs & AUV).<br>The record of the same data from two independent sensors ensures the consistency and reproducibility of measurements.
Randomization	Hydroacoustic and optic backscatter sensors were located in all geographical directions around the source
Blinding	n/a

Did the study involve field work? ☒ Yes ☐ No

Field work, collection and transport

Field conditions	Open-ocean conditions, please see: Vink, A. et al. MANGAN 2021 Cruise Report: Independent scientific monitoring of two collector tests in the BGR and GSR contract areas for the exploration of polymetallic nodules in the equatorial NE Pacific. BGR Report, 363 pp. (2022).
Location	Clarion-Clipperton Zone, eastern equatorial Pacific, outside of Exclusive Economic Zones, at 4500 m water depth.<br>Central coordinates: 14.112°N, 125.874°W
Access & import/export	We affirm that geological materials were collected and responsibly exported to our home laboratories, following relevant permits and local laws (no faunal samples were taken).

Disturbance	This study used hydroacoustic frequencies (2 MHz—400kHz) and optical wavelengths (700-880 nm) that are considered safe for marine organisms and are widely used in marine environmental studies.

Reporting for specific materials, systems and methods

We require information from authors about some types of materials, experimental systems and methods used in many studies. Here, indicate whether each material, system or method listed is relevant to your study. If you are not sure if a list item applies to your research, read the appropriate section before selecting a response.

Materials & experimental systems

n/a	Involved in the study
☒	☐ Antibodies
☒	☐ Eukaryotic cell lines
☒	☐ Palaeontology and archaeology
☒	☐ Animals and other organisms
☒	☐ Clinical data
☒	☐ Dual use research of concern
☒	☐ Plants

Methods

n/a	Involved in the study
☒	☐ ChIP-seq
☒	☐ Flow cytometry
☒	☐ MRI-based neuroimaging

Plants

Seed stocks	n/a
Novel plant genotypes	n/a
Authentication	n/a
